# Sociodemographic Determinants of Extreme Heat and Ozone Risk Among Older Adults in 3 Sun Belt Cities

**DOI:** 10.1093/geroni/igaf122.1353

**Published:** 2025-12-31

**Authors:** Peter Crank, Cassandra O’Lenick, Amir Baniassadi, David Sailor, Olga Wilhelmi, Mary Hayden

**Affiliations:** University of Waterloo, Waterloo, Ontario, Canada; The University of North Carolina at Chapel Hill, Chapel Hill, North Carolina, United States; Hebrew SeniorLife, Boston, Massachusetts, United States; Arizona State University, Tempe, Arizona, United States; National Center for Atmospheric Research, Boulder, Colorado, United States; University of Colorado Colorado Springs, Colorado Springs, Colorado, United States

## Abstract

**Background:**

Vulnerable populations across the United States are frequently exposed to extreme heat, which is becoming more intense due to a combination of climate change and urban-induced warming. Extreme heat can be particularly detrimental to the health and well-being of older citizens when it is combined with ozone. Although population-based studies have demonstrated associations between ozone, extreme heat, and human health, few studies focused on the role of social and behavioral factors that increase indoor risk and exposure among older adults.

**Methods:**

We conducted a household survey that aimed to understand how older adults are affected by extreme heat and ozone pollution inside and outside of their homes across Houston, Phoenix, and Los Angeles. We examine contributing factors to the risk of self-reported health effects using a generalized linear mixed-effects regression model of telephone survey data of 909 older adults in 2017.

**Results:**

We found an increased occurrence of self-reported symptoms for extreme heat with preexisting respiratory health conditions and a lack of air conditioning access; self-reported ozone symptoms were more likely with preexisting respiratory health conditions. The risk of heat-related symptoms was slightly higher in Los Angeles than Houston and Phoenix. We found several demographic, housing, and behavioral characteristics that influenced the risk of heat- and ozone-related symptoms.

**Conclusions:**

The increased risk among older adults based on specific social and behavioral factors identified in this study can inform public health policy and help cities tailor their heat and ozone response plans to the specific needs of this vulnerable population.

